# A Novel Interprofessional Mock Clinic Workshop for Medical Students With Orthotics and Prosthetics Students

**DOI:** 10.15766/mep_2374-8265.10836

**Published:** 2019-09-27

**Authors:** Sanghamitra M. Misra, Josh B. Utay, Larry Laufman

**Affiliations:** 1 Associate Professor, Department of Pediatrics, Baylor College of Medicine; 2 Assistant Professor, Orthotics and Prosthetics Program, School of Health Professions, Baylor College of Medicine; 3 Associate Professor, Department of Medicine, Baylor College of Medicine; 4 Associate Professor, Center for Medical Ethics and Health Policy, Baylor College of Medicine

**Keywords:** Orthotics, Prosthetics, O&P, Interprofessional Education, IPE, Musculoskeletal Exam, Physical Medicine and Rehabilitation, PM&R

## Abstract

**Introduction:**

Medical school education on orthotics and prosthetics (O&P) is limited, and O&P students receive limited education on performing comprehensive histories and physicals (H&Ps). This interprofessional workshop brings medical and O&P students together in a mock clinical setting. Students from one profession appraise the relationship of their scope/role to those of students of the other profession.

**Methods:**

Third-year medical students, second-year O&P students, and O&P patients participated in a 3-hour workshop. Students partnered into groups and rotated among stations performing patient history, physical exam, and O&P assessment. As a medical or O&P student completed the H&P, she or he explained the process to the student of the other profession. Each group assessed two patients and then presented one to a physical medicine and rehabilitation (PM&R) attending physician. The workshop concluded with a feedback session.

**Results:**

Immediate feedback was positive. Medical students, O&P students, PM&R physicians, and patients all commented favorably. In the workshop's first year, there were 19 responding students (10 medical, nine O&P); 68.4% said that the clinical session was better than expected, 73.7% were satisfied with the overall event, and 73.7% felt they were likely to use what they had learned in clinical practice. Feedback from learners included requesting more time for students to interact with each other after the mock clinic, sending preparation materials before the session, and focusing the medical student H&P on the musculoskeletal exam.

**Discussion:**

This workshop was well received by participants. Their feedback will help to continue and expand this collaboration.

## Educational Objectives

By the end of this activity, learners will be able to:

1. Identify one similarity and one difference between the medical history and the orthotics and prosthetics (O&P) history.
2. Identify one similarity and one difference between the medical physical exam and the O&P physical exam.
3. List two examples highlighting the importance of interprofessional collaboration in the care of patients with orthoses and prostheses.

## Introduction

Orthotics and prosthetics (O&P) is not a new field of medicine. Almost all physicians care for patients that use orthotic and/or prosthetic devices. However, current medical school education has limited curricula designed to expose medical students to the practice of O&P. General medical education for O&P students is also lacking in formal curricula. Although there is a wealth of evidence that interprofessional education (IPE) can be beneficial in medical education, to our knowledge there are no published data on O&P and medical school IPE. IPE occurs when students from two or more professions learn about, from, and with each other to enable effective collaboration and improve health outcomes.^1^ Our curriculum is true IPE. A 2018 meta-analysis concluded that educational intervention in various health care disciplines by means of an IPE program had a positive impact and was effective.^2^ Our session was intended to be IPE with interaction between the participants and focus on a goal, and not simply multiprofessional education, because the latter often does not result in appreciation for another's profession.^3^ Although our mock clinic is novel in the IPE literature, standardized patients have been used to create IPE sessions.^4^

For institutions that have both a medical school and an O&P program, coordinated IPE can enhance education for all students. Our targeted audience is O&P and medical students together. Considering that O&P is a very hands-on health care profession, a mock clinic with real O&P patients is a unique way of introducing medical students to the practice. O&P students are routinely taking care of complex medical patients, but these students have limited training in medical history taking and the comprehensive physical exam. O&P students can learn about the role of the physician from medical students. This curriculum was created as part of an ambulatory care course for third-year medical students. Previously, students received a lecture about the field of O&P. Although student feedback was positive, students consistently requested an experience where they could see and touch various orthoses and prostheses. This mock clinic workshop became an addition to the curriculum with continuation of the lecture on O&P. Considering that O&P patients are nearly impossible to simulate with standardized patients, we created a curriculum with real O&P patients in a mock clinic setting.

At this time, there are no curricula on *MedEdPORTAL* for O&P and medical student IPE. To our knowledge, there are no research studies examining medical students with O&P education. This is a unique clinical workshop and a novel contribution to the existing literature on *MedEdPORTAL*.

## Methods

This study was approved by the Baylor College of Medicine (BCM) Institutional Review Board (protocol number H-40763) on February 21, 2017.

BCM has both a medical school and an O&P program. The Longitudinal Ambulatory Course Experience (LACE) course for third-year BCM medical students introduced students to professionalism, bias, palliative care, and IPE. Through the LACE course, students worked alongside physical therapists, chaplains, music therapists, dental students, and many more health care professionals. The first introduction of O&P to LACE medical students was through one 2-hour lecture from O&P faculty. Because of consistent student feedback from the lecture requesting more interaction, this hands-on experience was created.

Considering that O&P patients are difficult to simulate with fellow students, or even with standardized patients, we created a mock clinic with real O&P patients so that medical students and O&P students could take histories, perform physical exams, write prescriptions, and offer guidance to real O&P patients. The mock clinic was conducted once yearly on the only date that all O&P second-year students were on-site at the medical school and the LACE course was in session for the medical students. At the time of this mock clinic, all of our medical students had completed classroom and clinical education on the musculoskeletal physical exam. For an ideal workshop experience, the medical students should have had education on the musculoskeletal physical exam. Other than this exception, there was no prerequisite knowledge or preparation needed for any of the participants in the session.

### Participants

1. Ten medical students were assigned to the mock clinic by a random LACE schedule generator.
2. Twenty second-year O&P students were assigned to the mock clinic.
3. Ten O&P patients were recruited for the workshop (five orthotics and five prosthetics).
4. Five physical medicine and rehabilitation (PM&R) BCM attending physicians were recruited to participate.

An email was sent to all medical and O&P students 1 week before the workshop to inform them of the upcoming IPE experience (Appendix A). In addition, a facilitator guide was sent to each of the participating PM&R physicians (Appendix B).

### Room Setup

The ideal setup was in a mock clinic with 10 standardized patient rooms, which provided adequate privacy for the patients and a quiet atmosphere for the learners. The first year we created this workshop, we held the session in a group lecture hall with exam beds along the walls. We had curtains separating patient beds from each other. The second year, we held the workshop within the O&P lab with beds set up along the walls and between O&P workbenches. The beds were far enough away from each other that we believed privacy was preserved. Although the sessions were loud at times, the students enjoyed observing their fellow students and their interactions with their patients. There was a great deal of interprofessional chatter, which is a wonderful part of IPE.

We asked all of our patient models to arrive at least 30 minutes before the workshop to make certain that they had sufficient time to walk over to the workshop area and get settled. We had water and snacks available for our patients. The patient models were reimbursed for their time at the standard college rate of $20 per hour.

### Schedule for the Workshop

• 00:00-00:15: Introduction, review of goals and objectives.
• 00:15-01:15: First patient assessment.
• 01:15-02:15: Second patient assessment.
• 02:15-03:15: Presentations to attendings.
• 03:15-03:30: Verbal feedback session.

At the start of our workshop, facilitators introduced themselves and had all participants introduce themselves. We then reviewed the goals and objectives and answered any questions (schedule, breaks, and closest restroom locations).

The students were then numbered and assigned to patients using the Mock Clinic Grid (Appendix C). Medical students and O&P students paired up and worked together. Medical students interviewed and examined each of their patients using the Musculoskeletal Exam Focused History and Physical Form (Appendix D). O&P students visited with each of their patients using the appropriate template: the Lower Limb Orthotics Prescription Recommendation Form (Appendix E), the Lower Limb Prosthetics Prescription Recommendation Form (Appendix F), the Upper Limb Orthotics Prescription Recommendation Form (Appendix G), or the Upper Limb Prosthetics Prescription Recommendation Form (Appendix H). During the history and physical (H&P), the student taking the H&P taught its specifics to the student of the other profession. Students were guided specifically to ask questions of each other to clarify their roles.

For the last hour of the workshop, medical students and their O&P student colleagues presented each of their patients to one of the PM&R attending physicians. The medical students reported their histories, physicals, assessments, and plans, and the O&P students reported their histories, physicals, and O&P prescription recommendations. The attending physicians gave valuable feedback on the histories taken and physical exams performed. Each attending physician spent 15 minutes with each group of students and continued to rotate among groups until all students completed their reports. While waiting for the faculty to come to their group, all students were encouraged to discuss their own professions and training programs with each other to enhance their understanding of each other's roles.

During the workshop conclusion, all participants (patient models, students, and attending physicians) were asked about the experience. All students were asked to complete a voluntary evaluation survey after the session (Appendix I). The survey was adapted from a standard course evaluation in the O&P program. The evaluation was used to modify the curriculum from year 1 to year 2.

## Results

During the first year of this workshop, 20 O&P students, 10 medical students, three orthotics patients, three prosthetics patients, two facilitators (one each from the School of Medicine and the O&P program), and two PM&R attending physicians participated.

During the second year of this workshop, 20 O&P students, 10 medical students, five orthotics patients, five prosthetics patients, two facilitators (again, one each from the School of Medicine and the O&P program), and five PM&R attending physicians participated.

The following changes were made in the curriculum from year 1 to year 2: (1) sending an introductory email with H&P templates to all participants, (2) revising the medical student H&P form to focus on the musculoskeletal system, (3) increasing the number of patients from six (three orthotics and three prosthetics) to 10 (five orthotics and five prosthetics), and (4) increasing the number of PM&R attending physicians.

This annual workshop included students of both medicine and O&P working together to care for O&P patients with the supervision of PM&R attending physicians in 2016 and 2017. The change in number of participants from year 1 to year 2 was due to enlarging the number of patients to increase exposure of students to O&P patients, as well as the number of supervising attending physicians to improve the flow of the clinic. Of the medical and O&P students who participated, 22 of the 30 students in 2016 and 16 of the 30 students in 2017 completed the postworkshop evaluation (Table 1). Overall, students of both kinds felt that the workshop provided a useful experience from which they learned relevant information. The mean score on satisfaction with the workshop was 2.77 on a scale from 1 (*extremely satisfied*) to 7 (*extremely dissatisfied*), indicating that the students felt the workshop was overall a good IPE experience (Table 2). As in most evaluations of curricula, verbatim comments can be very helpful. Considering from their comments that the 2016 students did not feel well prepared for this event, an introductory email was sent before the 2017 event, and those students felt that the workshop was well organized and that they were adequately prepared. The student comments have been helpful for improving the session from year to year. Some example comments follow.

**Table 1.** Workshop Students Who Completed Evaluations

	2016		2017		Total	
School	No.	%	No.	%	No.	%
Medical school	10	45.5	6	37.5	16	42.1
Orthotics & prosthetics	12	54.5	10	62.5	22	57.9
Total	22	100	16	100	38	100

**Table 2.** Course Evaluation Results

Question	*M*
Was this clinical experience what you expected? (1 = *a great deal better*, 7 = *a great deal worse*)	2.6
How useful was the information presented? (1 = *extremely useful*, 5 = *not at all useful*)	2.4
How prepared did you feel for this clinical experience? (1 = *extremely prepared*, 7 = *extremely unprepared*)	3.5
Overall, how satisfied were you with this event? (1 = *extremely satisfied*, 7 = *extremely dissatisfied*)	2.8
How likely will you use what you learned? (1 = *extremely likely*, 7 = *extremely unlikely*)	2.8

### Selected Verbatim Comments From Students in 2016

#### School of Medicine:

• “I do not know if every Med student will get this opportunity, but if not then students going into primary care should be higher priority for it. Going into ob/gyn, I doubt if I will ever write a prescription for orthotics or prosthetics.”
• “One change I would like to see in future programs is removing the extraneous history and physical information from the packet. Instead the med students should be told to do a focused history and physical. Just to prevent confusion.”
• “I believe more time for the med-students to talk to the O&P partners about O&P practice. My med-students had tons of questions and I felt it would have been helpful to have time to sit down and talk about these things and possibly take notes about what each other was saying.”
• “Was not [given] much of a heads up regarding what we would be doing beforehand. It would have been nice to receive an electronic copy of the handouts prior to the IPE session.”
• “Did not really know what was going to happen going in, but it was not an issue in terms of preparation.”
• “Very helpful given our limited exposure to O&P.”
• “It was interesting and good to see how the two specialties intersect.”
• “IPE experiences are always welcome and really help out in practice.”

#### School of O&P:

• “Just being given a more in-depth schedule of events would have been nice.”
• “Having participated in actual clinics on a regular basis made preparation a nonissue.”
• “I feel confident in my knowledge and skills to communicate with patients and fellow health care professionals.”
• “I was able to listen to the patient's history and see what I needed in the physical evaluation and that allowed me to provide a good recommendation.”
• “I felt like it was a great opportunity to educate medical students about what orthotists and prosthetists do.”
• “Pleased with the event but felt the interaction itself could be better if the med students were a bit more prepared.”

### Selected Verbatim Comments From Students in 2017

#### School of Medicine:

• “While I loved getting to meet the patients and hear their stories, I think the medical student learning experience could be improved by doing several smaller mock cases that focused more on the O&P aspect and less on the medical background. I think the medical student would benefit a lot more from learning major principles—a few basics types of orthotics or prosthetics and who benefits from each rather than delving in depth into only [two] cases where the information is so far beyond us.”
• “It had been a long time since I had performed a musculoskeletal exam, so I was initially a little worried about the clinical experience. However, the O&P students, standardized patients, and PM&R Attendings were all extremely friendly and helpful. Overall, the O&P clinic was extremely educational.”
• “Extremely well organized session; patients were amazing to work with [and] so were the O&P students and professors.”
• “I was not familiar with the O&P field before this experience and learned a lot from talking with the O&P students about their work.”

#### School of O&P:

• “I felt confident explaining to the medical students what we do.”
• “I felt I knew how to do an exam pertinent to OP care and was able to assist the medical exam in focusing on pertinent information.”
• “Overall was a good event. I feel [the] physical exam was important for us to see (O&P perspective), but a lot more time than we normally do. For the next time, I believe if the medical students could spend more time on [a] physical exam pertaining to our field, that would be helpful. For example, MMT [manual muscle testing] and ROM [range of motion] is relevant to us every day, but [the] condition of heart and lungs is not within our scope so we do not assess this.”
• “I thought it was very beneficial to work with the medical students and collaborate about a real patient to determine how to best meet their needs. It is often easy to get stuck in our own O&P world, and it is helpful to see the other aspects of the patient's care.”
• “I thought it was helpful but a little long for each patient encounter. I think it would be helpful to have [four] half hour encounters with an AFO [ankle-foot orthosis], KAFO [knee-ankle-foot orthosis], upper limb prosthesis and lower limb prosthesis. It would also be helpful to give the medical students a tour of the lab and different devices we fabricate/fit.”

## Discussion

This mock O&P clinic is an effective manner of exposing trainees from two related fields of health care to each other. This type of workshop has not been attempted previously and was relatively straightforward to plan. Finding adequate and appropriate space for the workshop was important to maintain confidentiality and privacy for the patients. However, utilizing large rooms with separations gave students the ability to see how their classmates were interacting with their respective patients. Considering that every O&P patient is unique, each student had much to learn. Although participants examined only one patient each, they were seeing interactions of students with all O&P patients. Our tremendously positive feedback from the sessions makes us certain that students will have a lasting impression from the workshop.

As mentioned previously, the first year we created this workshop, we held the session in a group lecture hall with exam beds along the walls. The second year, we held the workshop within the O&P lab. Although we believed that the second year setup would be a bit too crowded with O&P workbenches in the way, the medical students really enjoyed being in the lab and seeing some of the devices that O&P students had made and the tools used in the process. The O&P students enjoyed showing off some of their own creations as well.

We learned from the first year's verbal feedback session that students, mostly the medical students, felt unprepared for the session and did not know what to expect. To address that feedback, in the second year we sent an introductory email to all students 3 days before the workshop with an explanation of what would be expected. That email also had attachments of the various medical and O&P H&P forms that would be used during the session. During the second year's feedback session, everybody stated that they felt prepared for the workshop.

Patient models were thrilled to be part of this IPE experience. One patient commented in the group feedback session that “this was the best experience of my life.” They all agreed in group discussions that they wished their own physicians had experienced an educational curriculum like this one because their physicians rarely asked about or addressed their O&P needs.

Constructive feedback from learners also included (1) requesting more time for students to interact with each other after the session and (2) focusing the medical student H&P on the musculoskeletal exam. We made those changes by (1) leaving 15 minutes at the end of each H&P for students to answer questions and clarify specifics about the roles of the two professions and (2) creating a simplified medical student H&P. IPE can provide a valuable learning experience for all students involved. This particular workshop was impactful because of the interactive nature of the experience with real patients. The students were able to observe how the other profession approaches both patient interviews and physical exams. Seeing other students care for their patients with a different lens helped them understand the role of the other professional.

The main limitation of this resource is that only a program with both a medical school and an O&P program within the same institution (or at least in close proximity) could utilize the tool. In addition, this curriculum requires funding at least to compensate the O&P participating patient models appropriately for their time. Within our medical school curriculum, IPE is being expanded at the clerkship level, and this workshop could be incorporated into a clerkship so that more students are able to participate. There is also opportunity to incorporate more IPE-specific objectives and learn about student perceptions of various forms of IPE as the workshop is improved over time. It is important to note that this curriculum could also be adapted by a different combination of health care professionals to create an effective IPE workshop.

A second limitation of this curriculum is that knowledge was not assessed after the workshop. There is room for enhancement in the assessment of student knowledge for all students. The addition of questions after the session could include knowledge-based questions or ones specifically about whether objectives have been successfully reached.

### Conclusion

This interactive workshop with real patients was deemed to be a success by the facilitators, patient models, and most students in attendance. As IPE becomes more widely recognized as an essential aspect of the education of health care providers, this mock clinic can serve as a model for students of two (or more) synergistic professions to organize into a meaningful experience that meets numerous stated goals of IPE.

## Appendices

A. Letter to Medical and O&P Students.docx
B. Facilitator Guide for O&P IPE Workshop.docx
C. Mock Clinic Grid.xlsx
D. Musculoskeletal Exam Focused H&P Form.docx
E. LLO Rx Template.docx
F. LLP Rx Template.docx
G. ULO Rx Template.docx
H. ULP Rx Template.docx
I. O&P MS IPE Postworkshop Evaluation.docx All appendices are peer reviewed as integral parts of the Original Publication.
