# Supplementary material for: A Novel Interprofessional Mock Clinic Workshop for Medical Students With Orthotics and Prosthetics Students
Source: MedEdPORTAL. 2019 Sep 27;15:10836. doi: 10.15766/mep_2374-8265.10836 (PMC6869978; doi:10.15766/mep_2374-8265.10836)
Supplement: Supplementary file 1 — A. Letter to Medical and O&P Students.docx B. Facilitator Guide for O&P IPE Workshop.docx C. Mock Clinic Grid.xlsx D. Musculoskeletal Exam Focused H&P Form.docx E. LLO Rx Template.docx F. LLP Rx Template.docx G. ULO Rx Template.docx H. ULP Rx Template.docx I. O&P MS IPE Postworkshop Evaluation.docx [file mep-15-10836-s001.zip › A. Letter to Medical and O&P Students.docx]

Dear Medical and O&P students,

We are excited to have you participate in an interprofessional education (IPE) workshop next week on _______________. Medical students and orthotics and prosthetics (O&P) students will be brought together in a mock O&P clinic with real patients who use orthotic and/or prosthetic devices. IPE occurs when, “students from two or more professions learn about, from, and with each other to enable effective collaboration and improve health outcomes.” You will be expected to teach students from the other profession about your own and learn about patient care from the perspective of the other profession.

Please meet us promptly at 1:15 pm at the O&P classroom where we will conduct the workshop.

The format of the workshop:

1:15-1:30 Introduction, Review of Goals and Objectives

1:30-2:30 First Patient Assessment

2:30-3:30 Second Patient Assessment

3:30-4:30 Presentations to Attendings

Students are divided into groups consisting of one (third-year) medical student and two (second-year) O&P students. Each medical student will assess both patients: one in need of orthotic services, and the other in need of prosthetic services. Each O&P student in each group will take the lead on one patient encounter.

When assessing each patient, the medical student begins with a medical history specific to rehabilitation, orthopedic, or otherwise related aspects. This is followed by the O&P student evaluating for O&P services, including any device history. Students then take a moment to discuss any clarifying questions about history taking. Then, the medical student completes a focused musculoskeletal exam followed by the O&P student completing an O&P exam. Students again take a moment to ask each other any clarifying questions about the physical exams. Lastly, the medical student discusses the medical assessment and plan with the patient, and the O&P student presents the O&P assessment, plan, and O&P prescription recommendation with the patient and medical student.

When the faculty physicians arrive, both students (medical followed by O&P) present each of their patient histories, physical exams, assessments, and plans to the attending physician for both of their patients. Faculty may then share feedback and make recommendations concerning the process.

The objectives of the workshop:

By the end of the workshop, you will:

1. Identify one similarity and one difference between the medical history and the O&P history.

2. Identify one similarity and one difference between the medical physical exam and the O&P physical exam.

3. List two specific examples highlighting the importance of interprofessional collaboration and communication in the care of patients who use orthoses and prostheses.

Remember that interprofessional education (IPE) works best when all participants teach about their expertise and specialty and try to learn about the other discipline. Feel free to ask questions of other students during the workshop.

Sincerely,
Coordinators
