# Supplementary material for: A Novel Interprofessional Mock Clinic Workshop for Medical Students With Orthotics and Prosthetics Students
Source: MedEdPORTAL. 2019 Sep 27;15:10836. doi: 10.15766/mep_2374-8265.10836 (PMC6869978; doi:10.15766/mep_2374-8265.10836)
Supplement: Supplementary file 1 — A. Letter to Medical and O&P Students.docx B. Facilitator Guide for O&P IPE Workshop.docx C. Mock Clinic Grid.xlsx D. Musculoskeletal Exam Focused H&P Form.docx E. LLO Rx Template.docx F. LLP Rx Template.docx G. ULO Rx Template.docx H. ULP Rx Template.docx I. O&P MS IPE Postworkshop Evaluation.docx [file mep-15-10836-s001.zip › B. Facilitator Guide for O&P IPE Workshop.docx]

**Facilitator Guide for Physical Medicine and Rehabilitation (PM&R) Faculty**

**Orthotics and Prosthetics Interprofessional (IPE) Workshop**

Thank you for volunteering your valuable time to give your clinical guidance to our third-year medical and second-year O&P students in a mock clinic interprofessional workshop.

Workshop Essentials:

1. On the day of the workshop, the medical and O&P students will spend 2 hours working with each other and teaching each other about their own techniques in performing a history, physical, and appropriate prescriptions or guidance. They will do this by working with 2 patients per group (one patient using orthoses and one patient using prostheses).
2. You will arrive at the specified time in the calendar invite to give guidance to both medical and O&P students on the care of these special patients.
3. You will spend 15 minutes with each group of students and their assigned patient.
4. When you approach each group of students and their patient, start by establishing rapport with the patient.
5. The medical student in each group will present a focused history and physical related to the patient’s musculoskeletal and/or neurological condition and any guidance they offered.
6. The O&P student in each group will then present a focused O&P history and explain the O&P prescription recommendation.
7. Please offer feedback to the students on their history-taking skills and verbal presentations.
8. Please offer feedback to the students on proper physical exam techniques. You may ask the students to show you how they performed the physical exam or you can give feedback on verbal report of the exam.
9. Finally, please offer feedback on the medical and O&P recommendations
10. Try to save a few minutes at the end of each 15-minute session for question and answer with each group.
11. You will have the opportunity to rotate from group to group until all the students have been guided by a physician for both patients evaluated.
