# Supplementary material for: A Novel Interprofessional Mock Clinic Workshop for Medical Students With Orthotics and Prosthetics Students
Source: MedEdPORTAL. 2019 Sep 27;15:10836. doi: 10.15766/mep_2374-8265.10836 (PMC6869978; doi:10.15766/mep_2374-8265.10836)
Supplement: Supplementary file 1 — A. Letter to Medical and O&P Students.docx B. Facilitator Guide for O&P IPE Workshop.docx C. Mock Clinic Grid.xlsx D. Musculoskeletal Exam Focused H&P Form.docx E. LLO Rx Template.docx F. LLP Rx Template.docx G. ULO Rx Template.docx H. ULP Rx Template.docx I. O&P MS IPE Postworkshop Evaluation.docx [file mep-15-10836-s001.zip › D. Musculoskeletal Exam Focused H&P Form.docx]

**Musculoskeletal Exam Focused History and Physical Form**

Medical Student/Orthotics and Prosthetics Interprofessional Education (IPE) Workshop

**Chief Complaint (CC):**

**Past Medical History (PMH):** 

**Past Surgical History (PSH):** 

**Medications (MEDS):** 
*Includes all prescribed medications/over the counter medicines/non-traditional therapies. Note dosage/ frequency.*

**Allergies/Reactions (All/RXNs):** 

**Social History (SH):** 
Alcohol Intake/Cigarette smoking/tobacco use/Marital Status/Work History (type, duration, exposures)/Other (e.g. travel, pets, hobbies)

**Family History (FH):**

**Review of Systems (ROS):**

**Physical Exam:** 
Vital Signs: 
HEENT: Includes head, eyes, ears, nose, throat, oro-pharynx, thyroid. 
Lungs: 
Heart: 
Extremities (pulses/ROM/amputations) 
Neurologic:

- Motor Strength
- Sensation (light touch, pin prick, vibration and position)
- Reflexes, Babinski
- Cerebellar Function, Observed Ambulation

**Assessment and Plan:**
