# Supplementary material for: A Novel Interprofessional Mock Clinic Workshop for Medical Students With Orthotics and Prosthetics Students
Source: MedEdPORTAL. 2019 Sep 27;15:10836. doi: 10.15766/mep_2374-8265.10836 (PMC6869978; doi:10.15766/mep_2374-8265.10836)
Supplement: Supplementary file 1 — A. Letter to Medical and O&P Students.docx B. Facilitator Guide for O&P IPE Workshop.docx C. Mock Clinic Grid.xlsx D. Musculoskeletal Exam Focused H&P Form.docx E. LLO Rx Template.docx F. LLP Rx Template.docx G. ULO Rx Template.docx H. ULP Rx Template.docx I. O&P MS IPE Postworkshop Evaluation.docx [file mep-15-10836-s001.zip › E. LLO Rx Template.docx]

**Lower Limb Orthotics Prescription Recommendation Form**

Device:

- Name of Device & Side
- Custom-Fabricated vs. Custom-Fitted
- Construction
  - Conventional Metal & Leather
    - Aluminum vs. Stainless Steel
  - Thermoplastic
    - Kind & Thickness
- Articulations (if present)
  - Anatomical Joint Crossed
  - Brand / Type / Settings / Total ROM
- Interface
  - Padding: Globally vs. Regionally: _____________
  - Sock / Garment
    - Composition
    - Source (Specialized O&P vs. Generic)
- Closure
  - Straps vs. Shoes
- Shoe Recommendation
  - Type / Specifications:__________
  - Limitations of Shoe wear
    - Athletic Shoes Exclusively?
    - Heel Height Limits?

Wear & Care / Patient Education:

- Wear Schedule:
  - Break-in vs. Immediate Full-Time Use
  - Avg. Daily Use vs. Therapeutic
  - Duration: Prescribed vs. Anticipated
  - Wean Out Period (if necessary)
- Hygiene
  - Device
  - Patient
- Follow Up
  - Orthotist
  - Prescribing Physician
- Special Instructions
