# Supplementary material for: A Novel Interprofessional Mock Clinic Workshop for Medical Students With Orthotics and Prosthetics Students
Source: MedEdPORTAL. 2019 Sep 27;15:10836. doi: 10.15766/mep_2374-8265.10836 (PMC6869978; doi:10.15766/mep_2374-8265.10836)
Supplement: Supplementary file 1 — A. Letter to Medical and O&P Students.docx B. Facilitator Guide for O&P IPE Workshop.docx C. Mock Clinic Grid.xlsx D. Musculoskeletal Exam Focused H&P Form.docx E. LLO Rx Template.docx F. LLP Rx Template.docx G. ULO Rx Template.docx H. ULP Rx Template.docx I. O&P MS IPE Postworkshop Evaluation.docx [file mep-15-10836-s001.zip › F. LLP Rx Template.docx]

**Lower Limb Prosthetics Prescription Recommendation Form**

- Design
  - Post-Operative
  - Preparatory
  - Definitive
- Side
  - Left
  - Right
  - Bilateral
- Level
  - Syme’s / Transtibial
  - Knee Disartic. / Transfemoral
  - Hip Disartic. / HemiPelv.
- Construction
  - ENDOskeletal
  - EXOskeletal
- Socket Type
  - PTB / TSB
  - Ischial Containment / Quad / MAS / ??
  - Syme’s/KD Window Opening? Where?
- Layup
  - Standard
  - Heavy Duty
- Total Contact
  - ALWAYS
- Interface
  - Roll-on Liner
  - Foam Liner
  - Flexible Inner Socket
- Design
  - Post-Operative
  - Preparatory
  - Definitive
- Side
  - Left
  - Right
  - Bilateral
- Level
  - Syme’s / Transtibial
  - Knee Disartic. / Transfemoral
  - Hip Disartic. / HemiPelv.
- Construction
  - ENDOskeletal
  - EXOskeletal
- Socket Type
  - PTB / TSB
  - Ischial Containment / Quad / MAS / ??
  - Syme’s/KD Window Opening? Where?
- Layup
  - Standard
  - Heavy Duty
- Total Contact
  - ALWAYS
- Interface
  - Roll-on Liner
  - Foam Liner
  - Flexible Inner Socket
- Suspension
  - Leather Cuff
  - Anatomical (Condyles / Malleoli)
  - Knee Sleeve
  - Roll-on Locking Liner + Lock
  - Roll-on Suction Liner + Valve
  - Vacuum
  - Belt / Suspenders
- K-Level
  - K1, K2, K3, K4
- Knee
  - Friction / Single Axis
  - Poly-Centric
  - Hydraulic Modulated
  - Microprocessor-Modulated
- Foot / Feet
  - SACH
  - SAFE / Solid Ankle
  - Single Axis
  - Multi-Axis / Dynamic Resp
  - Syme’s / Low Clearance
  - Microprocessor-Modulated
  - Special Purpose
- Alignment
  - Standard
    - 5 skt flx (or 5 > knee flx contrctr.)
    - Inset foot 1/2" relative to socket; Look for vertical pylon at midstance
  - Special
- Cosmesis?
  - Color / Quality of Endo components
  - Silicone Restoration
  - Foam Cover & Skin
    - Pre-Fab vs. Custom?
    - Removable?
    - Water Proof?
- Options / Accessories
  - Shock-Absorbing Pylon?*
  - Torque Absorber?*
  - Waterproofing?
  - Growth Accommodations?
  - Cane / Walker / w/c leg rest?
  - Shoe / Car / Home Modifications?

*Ck for Ht. and K-Level

*Within foot or alone?

- Donning / Doffing of Device
  - Order of Operations
- Acceptable Shoes
  - Style
  - Size
  - Heel Ht.
- Wear of Device(s)
  - Break-in Period
    - Conditions?
  - Time of day?
  - Daily Use Max?
  - Shrinker use continued?
- Volume Mgmt. Considerations?
  - Socks, ½ socks, pads…
- Hygiene & Maintenance
  - Anatomical Limb
  - Interface
    - Liner / Sock Hygeine
  - Device
    - Socket, cover, components
- Follow Up
  - Prosthetist
    - Actual date
  - Prescribing MD
    - What circumstances?
- Special Instructions
