# Supplementary material for: A Novel Interprofessional Mock Clinic Workshop for Medical Students With Orthotics and Prosthetics Students
Source: MedEdPORTAL. 2019 Sep 27;15:10836. doi: 10.15766/mep_2374-8265.10836 (PMC6869978; doi:10.15766/mep_2374-8265.10836)
Supplement: Supplementary file 1 — A. Letter to Medical and O&P Students.docx B. Facilitator Guide for O&P IPE Workshop.docx C. Mock Clinic Grid.xlsx D. Musculoskeletal Exam Focused H&P Form.docx E. LLO Rx Template.docx F. LLP Rx Template.docx G. ULO Rx Template.docx H. ULP Rx Template.docx I. O&P MS IPE Postworkshop Evaluation.docx [file mep-15-10836-s001.zip › H. ULP Rx Template.docx]

**Upper Limb Prosthetics Prescription Recommendation Form**

Device:

- Design
  - Body-Powered vs. Passive vs. Externally-Powered
- Side
  - Left / Right / Bilateral
- Level
  - Partial Hand / Wrist-Disarticulation / Transradial / Transhumeral / Elbow Disartic. / Shoulder Disartic. / Scapulo-Thoracic
- Construction
  - ENDOskeletal vs. EXOskeletal
- Socket Type
  - Below Elbow: Short / Med / Long TR vs. Northwestern vs. Muenster
  - Above Elbow: Transhumeral vs. Frame + Flexible Inner Liner
  - Joint Disartic.: Self-Suspension
- Interface
  - Sock vs. Roll-on Gel Liner vs. Foam Liner vs. Flexible Inner Socket
- Suspension
  - Locking Liner vs. Suction vs. Supracondylar vs. Straps
- Elbow (if needed)
  - Flexible vs. Single/Poly Centric vs. Outside-Locking vs. Passive vs. MPC
- Terminal Device
  - Hook vs. Hand (General / Work / Pediatric / Passive / MPC)
  - Specific Use TD (Fork, Knife, Sport, Crawling, etc.)
- Cosmesis (if desired)
  - Standard Foam Cover & Skin vs. Silicone Restoration
- Options / Accessories
  - Quick-Disconnect Feature

Wear & Care, Patient Education:

- Donning & Doffing of Device
- Break-In Period
- Volume Mgmt. Considerations
- Hygiene & Maintenance
  - Anatomical Limb, Interface, Device
- Follow Up
  - Prosthetist
  - Prescribing MD
- Special Instructions
