# Supplementary material for: A Novel Interprofessional Mock Clinic Workshop for Medical Students With Orthotics and Prosthetics Students
Source: MedEdPORTAL. 2019 Sep 27;15:10836. doi: 10.15766/mep_2374-8265.10836 (PMC6869978; doi:10.15766/mep_2374-8265.10836)
Supplement: Supplementary file 1 — A. Letter to Medical and O&P Students.docx B. Facilitator Guide for O&P IPE Workshop.docx C. Mock Clinic Grid.xlsx D. Musculoskeletal Exam Focused H&P Form.docx E. LLO Rx Template.docx F. LLP Rx Template.docx G. ULO Rx Template.docx H. ULP Rx Template.docx I. O&P MS IPE Postworkshop Evaluation.docx [file mep-15-10836-s001.zip › I. O&P MS IPE Postworkshop Evaluation.docx]

**O&P and MS IPE Post Workshop Evaluation**

1. Which school are you in?
   1. Medical school
   2. O&P school
2. Was this clinical experience better than what you expected, worse than what you expected, or about what you expected?
3. A great deal better
4. Quite a bit better
5. Somewhat better
6. About what was expected
7. Somewhat worse
8. Quite a bit worse
9. A great deal worse
10. How useful was the information presented at this event?
    1. Extremely useful
    2. Moderately useful
    3. Quite useful
    4. Slightly useful
    5. Not useful
11. Comments: please elaborate on your reply to the previous question
12. How prepared did you feel for this clinical experience?
    1. Extremely prepared
    2. Moderately prepared
    3. Somewhat prepared
    4. Neutral
    5. Somewhat unprepared
    6. Moderately unprepared
    7. Extremely unprepared
13. Overall, were you satisfied with this event, dissatisfied with this event, or neither satisfied nor dissatisfied?
    1. Extremely satisfied
    2. Quite satisfied
    3. Somewhat satisfied
    4. Neither satisfied nor dissatisfied
    5. Somewhat dissatisfied
    6. Quite dissatisfied
    7. Extremely dissatisfied
14. Comments: Please elaborate on your reply to the previous question
15. How likely are you to use what you learned here in your clinical practice?
    1. Extremely likely
    2. Moderately likely
    3. Somewhat likely
    4. Neither likely nor unlikely
    5. Somewhat unlikely
    6. Moderately unlikely
    7. Extremely unlikely
16. Comments: Please elaborate on your reply to the previous question.
17. We welcome your feedback! Please convey any questions, comments or concerns below. Thank you.
